# Inhibition of CCL2 by bindarit alleviates diabetes-associated periodontitis by suppressing inflammatory monocyte infiltration and altering macrophage properties

**DOI:** 10.1038/s41423-020-0500-1

**Published:** 2020-07-16

**Authors:** Zongshan Shen, Shuhong Kuang, Min Zhang, Xin Huang, Jiayao Chen, Meiliang Guan, Wei Qin, Hockin H. K. Xu, Zhengmei Lin

**Affiliations:** 1 Hospital of Stomatology, Guangdong Provincial Key Laboratory of Stomatology, Guanghua School of Stomatology, Sun Yat-sen University, Guangzhou, Guangdong China; 2 Department of Andrology, The First Affiliated Hospital, Sun Yat-sen University, Guangzhou, Guangdong China; 3 The Key Laboratory for Stem Cells and Tissue Engineering, Center for Stem Cell Biology and Tissue Engineering, Ministry of Education, Sun Yat-sen University, Guangzhou, Guangdong China; 4 Department of Advanced Oral Sciences & Therapeutics, University of Maryland School of Dentistry, Baltimore, MD USA; 5 Center for Stem Cell Biology and Regenerative Medicine, University of Maryland School of Medicine, Baltimore, MD USA; 6 University of Maryland Marlene and Stewart Greenebaum Cancer Center, University of Maryland School of Medicine, Baltimore, MD USA

**Keywords:** Diabetes-associated periodontitis, bindarit, proinflammatory monocytes, macrophages, Chemokines, Chronic inflammation

## Abstract

Diabetes-associated periodontitis (DP) aggravates diabetic complications and increases mortality from diabetes. DP is caused by diabetes-enhanced host immune-inflammatory responses to bacterial insult. In this study, we found that persistently elevated CCL2 levels in combination with proinflammatory monocyte infiltration of periodontal tissues were closely related to DP. Moreover, inhibition of CCL2 by oral administration of bindarit reduced alveolar bone loss and increased periodontal epithelial thickness by suppressing periodontal inflammation. Furthermore, bindarit suppressed the infiltration of proinflammatory monocytes and altered the inflammatory properties of macrophages in the diabetic periodontium. This finding provides a basis for the development of an effective therapeutic approach for treating DP.

## Introduction

Diabetes often enhances the chronic inflammation of various organs, including the kidneys, heart, and periodontium.^1,2^ As one of its most prevalent complications, diabetes-associated periodontitis (DP) causes lesions in the periodontium, including the gingiva, periodontal ligament and alveolar bone, which can lead to tooth loss.^2^ Additionally, periodontal disease increases the risk of various systemic diseases such as diabetes,^3^ atherosclerosis^4^, and Alzheimer’s disease.^5^ Current standard treatments for periodontitis include scaling and root planing, sometimes combined with antimicrobials. These treatments usually do not completely eliminate periodontal pathogens.^6^ Diabetes enhances the host inflammatory response to periodontal pathogens.^7^ DP patients exhibit marked alveolar bone loss and a high recurrence rate after routine treatment.^8,9^ Therefore, there is an urgent need to investigate an alternative strategy for DP treatment.

Dysregulation of the host immune-inflammatory response, rather than the direct effects of bacterial infection, ultimately induces periodontium destruction.^6,10^ Specifically, proinflammatory cytokines such as IL-1 and TNF-α are responsible for periodontal inflammation and alveolar bone resorption.^11,12^ Monocytes are an important source of proinflammatory cytokines in the periodontitis-affected periodontium.^13,14^ Diabetes can lead to prolonged monocyte infiltration into inflamed sites by activating precursors in the bone marrow.^13,15^ Excessive monocyte infiltration contributes to persistent tissue inflammation and increases the severity of diabetic complications due to the release of inflammatory mediators such as TNF-α, IL-1β, and IL-6 by these cells.^16,17^ Monocytes also release matrix metalloproteinases (MMPs), which cause tissue destruction by degrading the extracellular matrix.^18^ Chemokine-chemokine receptor signaling results in the recruitment of monocytes to the inflamed site.^19^ CC chemokine ligand 2 (CCL2) can modulate monocyte recruitment in multiple inflammatory diseases by interacting with its corresponding receptor, CCR2, which is present on monocytes.^20^ Reducing monocyte recruitment to inflamed sites via the inhibition of CCL2 has been reported to effectively suppress inflammation in many mouse models. For example, the inhibition of CCL2 signaling alleviates peripheral neuropathy,^21^ osteoarthritis^22^, and liver injury^23^ by reducing monocyte infiltration in mice. However, it remains unclear whether CCL2 inhibition is an effective strategy to treat DP.

CCL2 levels are elevated in gingival biopsies and in serum from patients with periodontitis, and elevated CCL2 levels have been reported to be associated with persistent periodontal inflammation.^24,25^ Fibroblasts, monocytes, macrophages, and endothelial cells have been reported to be the main producers of CCL2 in the periodontium.^26,27^ Under diabetic conditions, CCL2 levels were persistently upregulated in inflamed gingiva.^28,29^ CCL2 derived from inflamed gingiva could be involved in the recruitment of monocytes from the peripheral circulation into periodontal tissues, leading to persistent periodontal inflammation.^13,30^ Additionally, CCL2 has been reported to enhance osteoclastogenesis by inducing the expression of receptor activator of NF-κB (RANK) by osteoclast progenitors.^31^ RANK ligand (RANKL) further promotes the differentiation of osteoclast progenitors into functional osteoclasts, which could aggravate DP by causing alveolar bone resorption.^32^ These findings suggest that CCL2 inhibition could be a potential therapeutic strategy to effectively treat DP.

Hence, we developed an experimental model of periodontitis in diabetic mice that can mimic the state of persistent CCL2 elevation in patients with DP. Bindarit, a CCL2 synthesis inhibitor, was selected for use in this study because it has been shown in phase II clinical trials to have favorable safety and tolerability profiles.^33^ Bindarit has been reported to reduce CCL2 expression and attenuate inflammation in various inflammatory diseases in mouse models of diseases such as osteoarthritis^22^ and peripheral neuropathy.^21^ The mechanism of CCL2 inhibition has been shown to involve the inhibition of p65- and p65/p50-induced CCL2 promoter activation.^34^ The therapeutic effects of bindarit are related to its ability to suppress monocyte recruitment. Thus, bindarit could be a promising drug for the treatment of DP.

## Methods and materials

### Mice

Male C57BSK-*db/db* mice and male C57BL/6 mice were purchased from the National Resource Center of Model Mice (Nanjing, China). C57BSK-*db/db* mice are widely accepted as an animal model of diabetes. All experiments were performed at the Laboratory Animal Center of Sun Yat-sen University and approved by the Animal Care and Use Committee of Sun Yat-sen University (SYSU-IACUC-2018-000135). Eight- to sixteen-week-old male mice were used in all experiments unless otherwise noted.

### Animal experiments

A mouse model of periodontitis was established as previously described.^35,36^ Briefly, a mouse was placed in a sealed container with a 4% (vol/vol) isoflurane flow until full anesthesia was achieved. A 5-0 silk ligature was tied around the maxillary left second molar of the isoflurane-anaesthetized mouse. The ligature was removed 10 days after placement. The mice were sacrificed on the indicated days (Supplementary Fig. 1).

To investigate the therapeutic effects of a CCL2 inhibitor (bindarit) on DP in *db/db* mice, the mice were randomly divided into two groups: a 0.5% carboxymethylcellulose (CMC; vehicle)-treated group and a bindarit-treated group (6 animals per group at each time point). Bindarit (50 mg/kg) or vehicle was orally administered daily from day 0 to day 28 after ligature removal. The mice were sacrificed on day 28 after ligature removal. Micro-CT and histological analyses were used to assess periodontitis-induced bone loss and inflammation severity. The evaluations were conducted by three investigators who were blinded to the grouping information.

For histological analyses, maxillae fixed in 4% paraformaldehyde (PFA) were decalcified in 0.5 M EDTA for 2 weeks, embedded in Tissue-Tek optimum cutting temperature (OCT) compound (Sakura Finetek, Torrance, CA, USA) and frozen in a cryostat (Leica CM1900, Germany). Sections were stained with hematoxylin and eosin (H&E) and tartrate-resistant acid phosphatase (TRAP; Jiancheng Technology, Nanjing, China). TRAP-positive multinucleated cells (>3 nuclei) were considered osteoclasts. The distance between the cementoenamel junction (CEJ) and the alveolar bone crest (ABC) was measured to evaluate bone loss as previously described.^36^

For microcomputed tomography (micro-CT) analysis, the collected maxillae were fixed in 4% PFA for 24 h, washed in 1× phosphate-buffered saline (PBS), dehydrated in 75% ethanol, placed in standardized cylindrical sample holders and scanned by high-resolution micro-CT (Scano Medical AG, Bassersdorf, Switzerland). The key parameters were as follows: 70 kV, 114 mA, 20 µm increments, and a 3000-ms integration time. Image data were reconstructed and analyzed using three-dimensional image analysis software (VGStudio MAX 1.2.1, Heidelberg, Germany). The CEJ-ABC distance was measured as previously described.^36^

### Immunofluorescence (IF) analysis

Maxillae were embedded in Tissue-Tek OCT compound and frozen in a cryostat (Leica CM1900, Germany). Sections (10 μm thick) were prepared, blocked with 0.05% PBS-Tween containing 0.5% fetal bovine serum (FBS) for 30 min (min), and incubated sequentially with primary antibodies or an IgG isotype control antibody overnight at 4 °C and with secondary antibodies for 60 min at room temperature. The sections were then counterstained with 4′,6-diamidino-2-phenylindole (DAPI) for 3 min at room temperature. IF signals were visualized and recorded using a laser scanning confocal microscope (LSM780, Zeiss, Germany).

### Tissue extraction and single-cell preparations

Blood was extracted from the tail veins of fully anaesthetized mice. The mice were then sacrificed, and the periodontium was collected. Single-cell suspensions were generated from tissues as described below.

The procedure for obtaining single-cell suspensions from gingival tissues was modified from the previously described method.^37^ Briefly, upper gingival tissues were collected and cut into small fragments. The fragments were incubated in RPMI 1640 medium (Gibco; Thermo Fisher Scientific, Inc., Waltham, MA, USA) containing 3 mg/ml collagenase type I (Sigma-Aldrich, Saint Louis, MO, USA) and 4 mg/ml dispase (Sigma-Aldrich) at 37 °C for 60 min. The treated material was filtered through a stainless-steel filter (mesh size 70 μm) to remove tissue fragments and resuspended in fresh RPMI medium.

Blood cells were processed by the following method. A total of 500 μL of blood was collected into tubes containing a 10% (vol/vol) solution of 0.5 M EDTA. The blood samples were subjected to red blood cell (RBC) lysis using RBC lysis buffer (Kangwei, Jinzhong, China) according to the manufacturer’s instructions. The remaining cells were centrifuged (5 min at 450×*g*), the supernatant was discarded, and the pellet was resuspended in PBS.

### Flow cytometry and fluorescence-activated cell sorting (FACS)

The single-cell suspensions were resuspended in Zombie viability dye at 10^5^–10^6^ cells/100 μl (BioLegend, San Diego, CA, USA) and incubated for 15 min at room temperature to enable the exclusion of dead cells from the analysis. For surface antigen staining, the cells were incubated first with Fc blocker (BioLegend) at 4 °C for 10 min and then with the appropriate antibody in the dark at 4 °C for 30 min. For intracellular antigen staining, cell surface antigens were first stained; the cells were then fixed in fixation buffer (0.5 ml/tube; BioLegend) in the dark for 20 min and centrifuged at 350×*g* for 5 min, and the supernatant was discarded. A predetermined antibody of interest was added, and the samples were incubated in the dark at 4 °C for 20–30 min. The cells were washed, resuspended and analyzed by flow cytometry (FACScan; Becton Dickinson, San Diego, CA, USA). FACS was performed in a MoFlo Astrios EQs (Beckman Coulter, USA). The purity of the sorted CD45^+^F4/80^+^ cells was evaluated by flow cytometry (Supplementary Fig. 5). The gating strategies used for the flow cytometric analysis of periodontal cells are shown in Supplementary Fig. 4, and the gating strategies used for the flow cytometric analysis of blood cells are shown in Supplementary Fig. 6. The gating strategies used for the FACS of periodontal tissue samples are shown in Supplementary Fig. 4. At least three independent experiments were performed for each analysis. The data were analyzed using FlowJoV10.0 (Tree Star, Ashland, OR, USA).

### RNA isolation, reverse transcription, and RT-qPCR

Total RNA was purified from periodontal tissue with TRIzol reagent (Invitrogen, Carlsbad, CA, USA), and cDNA was generated using PrimeScript RT Master Mix (Toyobo Co, Ltd, Osaka, Japan). The cells isolated by FACS were lysed, and reverse transcription was performed using a SYBR™ Green Fast Advanced Cells-to-CT™ Kit (Thermo Fisher Scientific, Inc., Waltham, MA, USA). Gene expression levels were measured by RT-qPCR using a BioRad CFX96^TM^ Detection System (Roche, Sweden) and SYBR PCR Master Mix (Roche, Indianapolis, IN, USA). The primers used in this experiment are shown in Supplementary Table 2.

### RNA sequencing analysis

The periodontium was extracted from three *db/db* mice on day 10 after ligature and from three *db/db* mice without ligature as controls. RNA was isolated from the periodontium using TRIzol reagent. RNA sequencing libraries were constructed using an NEBNext^®^ Ultra™ RNA Library Prep Kit and were then subjected to deep sequencing via the Illumina Sequencing Platform (HiSeq, Fasteris SA, Switzerland) at RiboBio Co. Ltd., Guangzhou, China.

Bioconductor was used to analyze the raw gene count matrix. FastQC was performed as a quality control of the raw sequencing data. Differentially expressed genes (DEGs) were analyzed using the edgeR analysis package in the R statistical program with the criteria of an adjusted *p* value of ≤0.05 and an absolute log2(fold change) of >2. RStudio was used to create heat maps and volcano plots. Gene Ontology (GO) functional analysis was performed for the top 200 upregulated DEGs using the Database for Annotation, Visualization and Integrated Discovery (DAVID). The DAVID enrichment table is shown in Supplementary Table 1.

### Western blot analysis

Proteins were extracted from cells and tissues using radioimmunoprecipitation assay (RIPA) buffer (Millipore, MA, USA) for 30 min on ice. The concentration of total protein in RIPA-extracted lysates was measured using a bicinchoninic acid (BCA) protein assay kit (CWBioTech, Beijing, China). SDS-PAGE was performed to separate the proteins, which were then transferred to a polyvinylidene fluoride membrane (PVDF; Millipore, MA, USA). Bovine serum albumin (5%) was used to block the PVDF membranes for 30 min at room temperature. The membranes were incubated with the indicated primary antibodies at 4 °C overnight and then incubated with secondary antibodies conjugated to horseradish peroxidase for 1 h at room temperature. A chemiluminescence kit (Millipore, MA, USA) was used to detect the target bands. Information on the antibodies used in these experiments is provided in Supplementary Table 3.

### ELISA and cytokine measurements

The concentrations of cytokines in mouse serum, gingival tissue, cell culture medium and cell lysates were measured using CCL2 (RayBiotech, Norcross, GA, USA), TNF-α (CUSABIO, Wuhan, China) and IL-1β (CUSABIO) ELISA kits according to the manufacturers’ instructions.

A LEGENDplex^TM^ mouse inflammation panel (BioLegend) was used to measure the concentrations of 13 individual cytokines in tissue culture supernatants, serum samples, and RIPA-extracted lysates according to the manufacturer’s instructions.^38^ The extracts were concentrated to obtain the proper concentration using vacuum freeze-drying equipment (Songyuan, Beijing, China). The concentration of total protein in RIPA-extracted lysates was measured using a BCA protein assay kit, and equal amounts of total protein were used in the following assay. The kit provides capture beads that are conjugated to specific antibodies, rendering them easily distinguishable by size and fluorescence signal. The biological samples were incubated with the capture beads at room temperature for 2 h. A biotinylated detection antibody was then added, and the samples were subjected to flow cytometry. LEGENDplex8.0 data analysis software was used to calculate the mean fluorescence intensity (MFI) corresponding to each cytokine. Cytokine concentrations were measured by comparing the fluorescence intensity to that of a standard.

### Ex vivo stimulation and cytokine measurements

Periodontal macrophages and blood monocytes were isolated, and 2500 isolated cells were incubated in 50 μl of RPMI medium in individual wells of 384-well culture plates. The maxillary gingival tissues from four mice were pooled to obtain this cell density. For the stimulation experiment, 100 ng/ml lipopolysaccharide (LPS; Sigma-Aldrich) was added to the culture medium, and the culture supernatants were collected after 24 h. For the bindarit experiment, isolated cells were pretreated with 25 μM, 100 μM or 400 μM bindarit for 1 h and then stimulated with 100 ng/ml LPS for 24 h prior to collection of the culture supernatant.

For intracellular cytokine analysis by flow cytometry, isolated periodontal cells were incubated in RPMI medium supplemented with 100 ng/ml LPS. After 6 h, cytokine secretion was blocked by the addition of 10 µg/ml brefeldin-A (Invitrogen eBioscience, USA) for 2 h at 37 °C. The cells were then prepared for staining and flow cytometric analysis.

### Statistics

Comparisons between groups were performed using an unpaired two-tailed Student’s *t* test or one-way analysis of variance (ANOVA) with Tukey’s multiple comparison test. Error bars indicate the standard error of the mean. *P* < 0.05 was considered to indicate statistical significance. All statistical analyses were performed with Prism software (GraphPad).

## Results

### CCL2 levels are persistently elevated in DP mice

**Fig. 1:**
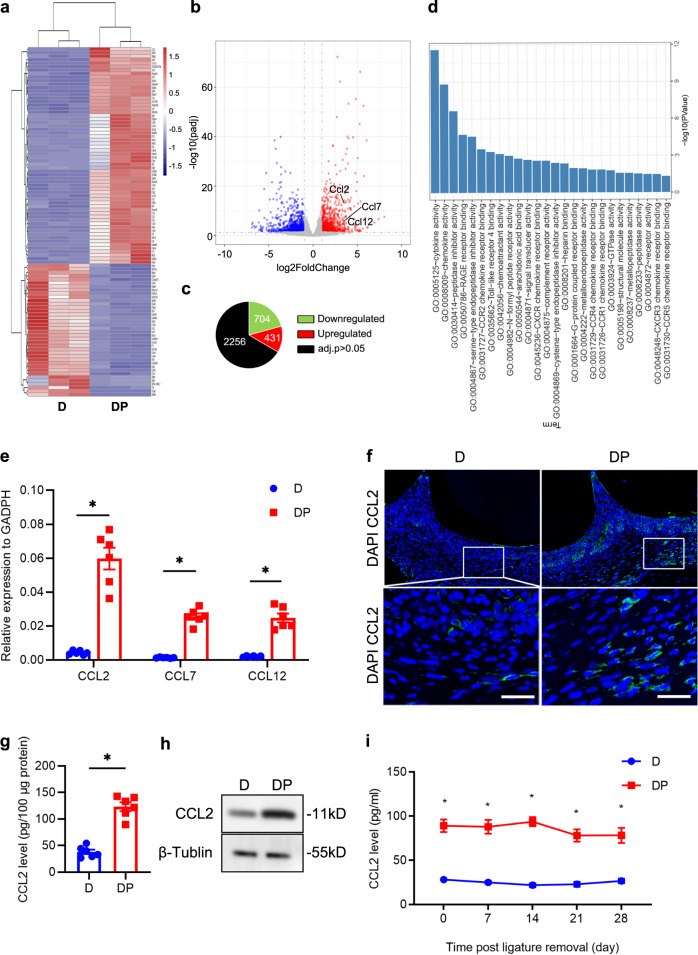
CCL2 levels are persistently elevated in DP mice. **a** Heat map showing the differentially expressed genes in the periodontium of diabetic mice with periodontitis (DP mice, *n* = 3) vs. the periodontium of diabetic mice without periodontitis (D mice, *n* = 3). **b** Volcano plots showing DEGs. **c** Pie chart showing the number of dysregulated genes. **d** Gene Ontology (GO) functional analysis of the top 200 upregulated genes in the periodontium of DP mice. **e** The mRNA levels of CCL2, CCL7, and CCL12 in the periodontium of DP mice (*n* = 6) and D mice (*n* = 6) 10 days after ligature placement were analyzed by RT-qPCR. Error bars indicate SEM. *p < 0.05 (unpaired two-tailed Student’s *t* test). **f** CCL2 expression in the periodontium of each group (*n* = 6 per group) was analyzed by IF staining 10 days after ligature placement. Nuclei were stained with DAPI. Scale bar = 25 μm. **g** CCL2 levels in cells extracted from the periodontium of each group (*n* = 6 per group) were analyzed by ELISA 10 days after ligature placement. Error bars indicate SEM. *p < 0.05 (unpaired two-tailed Student’s *t* test). **h** CCL2 levels in the periodontium of each group were measured by western blot analysis 10 days after ligature placement (*n* = 6 per group). **i** Serum levels of CCL2 in each group were analyzed by ELISA on days 0, 7, 14, 21, and 28 after ligature removal (ligature-induced periodontitis mode, *n* = 6 per group at the indicated time points). Error bars indicate SEM. *p < 0.05 (unpaired two-tailed Student’s *t* test).

To investigate the mechanisms responsible for severe inflammation in periodontitis under diabetic conditions, we performed RNA sequencing analysis of periodontium from diabetic *db/db* mice with periodontitis (DP mice) and diabetic *db/db* mice without periodontitis (D mice). Approximately 1130 genes were dysregulated in the periodontium of DP mice compared with that of D mice (Fig. 1a–c). We conducted GO functional analysis of the top 200 upregulated DEGs based on biological process. GO term enrichment analysis showed that the upregulated DEGs were significantly associated with cytokine/chemokine activity and CCR2 chemokine receptor binding (Fig. 1d, Supplementary Table 1). CCR2 ligands, including CCL2, CCL7, and CCL12, were highly upregulated in DP mice (Fig. 1b). RT-qPCR analysis further confirmed that CCL2, CCL7, and CCL12 mRNA expression levels were significantly increased in the periodontal tissues of DP mice compared with those of D mice. On day 10 after ligature, the expression of CCL2 was the highest among the chemokines examined (Fig. 1e). The high CCL2 level in the inflamed periodontium of diabetic mice was further confirmed by IF, ELISA and western blot assays (Fig. 1f–h, and Supplementary Fig. 2a). We also measured the expression of CCL2 in the periodontium of nondiabetic mice with periodontitis (NP mice) and without periodontitis (N mice). RT-qPCR analysis showed that CCL2 expression was upregulated in the periodontium of NP mice on day 10 after ligature. The upregulation of CCL2 expression in the inflamed periodontium of NP mice was further confirmed by ELISA (Supplementary Fig. 3a, b).

To investigate the kinetics of CCL2 production after the induction of periodontitis in diabetic and nondiabetic mice, we measured serum CCL2 levels in D and DP mice at various times (Supplementary Fig. 1). The ELISA results indicated that CCL2 levels remained high for at least 4 weeks after ligature removal (Fig. 1i). We also measured serum CCL2 levels in N and NP mice. ELISA showed that serum CCL2 levels decreased dramatically 6 days after ligature removal in NP mice (Supplementary Fig. 3c). Taken together, these results indicate that CCL2 levels are briefly upregulated in NP mice but are persistently elevated in DP mice.

### Proinflammatory monocytes persistently infiltrate the periodontium of DP mice

**Fig. 2:**
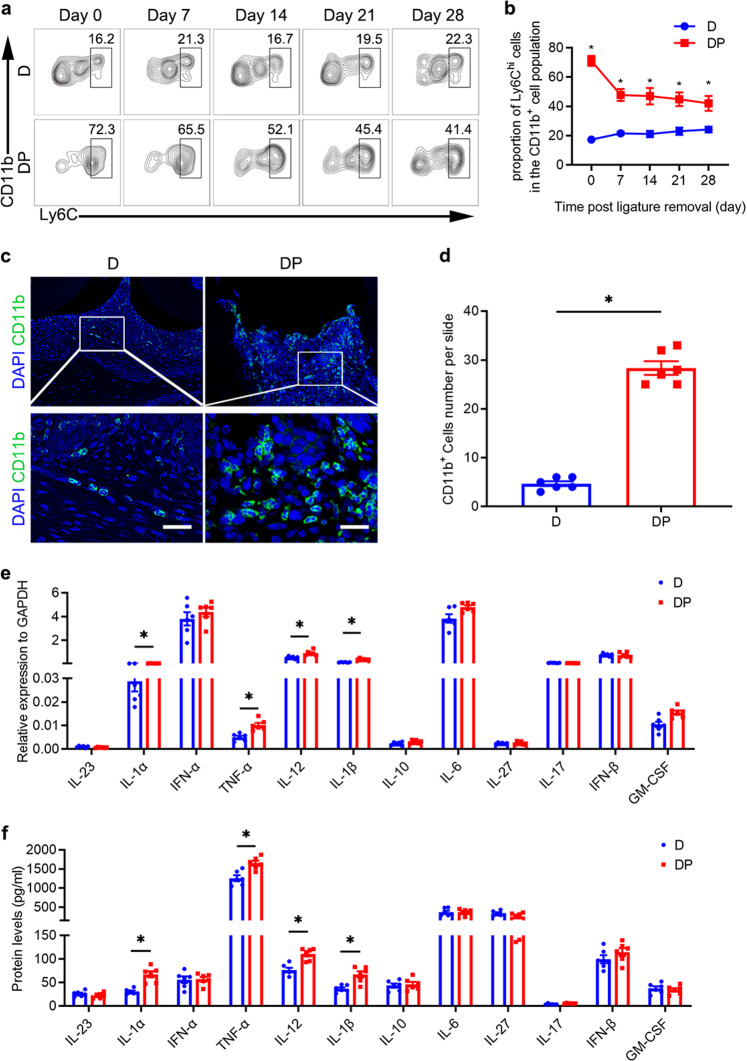
Proinflammatory monocytes persistently infiltrate the periodontium of DP mice. **a** The proportion of Ly6C^hi^ cells in the CD11b^+^ cell population in the periodontium of DP and D mice was analyzed by flow cytometry on days 0, 7, 14, 21, and 28 after ligature removal (*n* = 6 per group at each time point). Gates captured single, live Lin [CD3, CD19, NK1.1, Ly6G]^–^CD11b^+^ cells in the periodontium. **b** Statistical analysis of flow cytometry data showing the proportion of periodontal Ly6C^hi^ cells in an individual, live Lin^-^CD11b^+^ cell population in each group (*n* = 6 per group at each time point). Error bars indicate SEM. *p < 0.05 (unpaired two-tailed Student’s *t* test). **c** CD11b^+^ cells in the periodontium of each group (n = 6 per group) were analyzed by IF staining on day 28 after ligature removal. Nuclei were stained with DAPI. Scale bar = 25 μm. The number of CD11b^+^ cells in each microscopic field of view was quantified. Error bars indicate SEM. *p < 0.05 (unpaired two-tailed Student’s *t* test). **d** Statistical analysis of the IF staining of periodontal CD11b^+^ cells in each group (n = 6 per group). Error bars indicate SEM. *p < 0.05 (unpaired two-tailed Student’s *t* test). **e** The mRNA expression levels of various inflammatory cytokines in CD45^+^F4/80^+^ cells isolated from the periodontium of each group (*n* = 6 per group) were analyzed by RT-qPCR on day 28 after ligature removal. Error bars indicate SEM. *p < 0.05 (unpaired two-tailed Student’s *t* test). **f** The levels of various inflammatory cytokines in the culture supernatant of CD45^+^F4/80^+^ cells isolated from each group (*n* = 6 per group) were analyzed by LEGENDplex^TM^ bead-based immunoassays on day 28 after ligature removal. Error bars indicate SEM. *p < 0.05 (unpaired two-tailed Student’s *t* test).

CCL2-mediated proinflammatory monocyte infiltration is a critical process in multiple inflammation-associated diseases.^22,39^ CD11b^+^Ly6C^hi^ cells are widely considered to be proinflammatory monocytes.^16,40^ Flow cytometric analysis showed that the proportion of Ly6C^hi^ cells in the periodontal tissues of DP mice was significantly higher than the proportion of such cells in the periodontal tissues of D mice from day 7 to day 28 after ligature removal (Fig. 2a, b; Supplementary Fig. 4). IF analysis confirmed that significantly more CD11b^+^cells were present in the periodontal tissues of DP mice than in those of D mice on day 28 after ligature removal (Fig. 2c, d and Supplementary Fig. 2b). We also investigated the proportional change in Ly6C^hi^ cells in the periodontal tissues of NP and N mice from day 0 to day 9 after ligature removal. Flow cytometric analysis showed that the proportion of Ly6C^hi^ cells in the periodontal tissues of NP mice decreased by approximately 65% between day 0 and day 9 after ligature removal (Supplementary Fig. 3d, e).

Accumulating evidence indicates that infiltration by proinflammatory monocytes impacts the properties of tissue macrophages.^41^ These macrophages could participate in the progression of periodontitis by releasing inflammatory cytokines such as IL-1β, IL-6, and TNF-α.^42,43^ CD45^+^F4/80^+^ cells are considered macrophages in the periodontium.^42,43^ Hence, we used flow cytometry to isolate CD45^+^F4/80^+^ cells from the periodontal tissues of the experimental mice on day 28 after ligature removal. The purity of the sorted CD45^+^F4/80^+^ cells was 96.0% (Supplementary Fig. 5a, b). RT-qPCR analysis showed that higher mRNA levels of the inflammatory cytokines IL-1α, TNF-α, IL-12, and IL-1β were present in cells isolated from DP mice than in cells isolated from D mice. Notably, the level of TNF-α mRNA was approximately 2.1-fold higher in isolated macrophages from DP mice than in macrophages from D mice (Fig. 2e). Isolated macrophages were cultured and stimulated with 100 ng/ml LPS, and the culture supernatant was collected for the analysis of inflammatory cytokine levels after 24 h. The results of LEGENDplex^TM^ bead-based immunoassays indicated that IL-1α, TNF-α, IL-12, and IL-1β levels increased by approximately 2.2-fold, 1.3-fold, 1.4-fold, and 1.8-fold, respectively, in the macrophage supernatant of the DP group compared with that of the D group (Fig. 2f). These results demonstrate that persistent infiltration by proinflammatory monocytes is related to DP.

### Inhibition of CCL2 production by bindarit rescues epithelial lesions and alveolar bone loss in DP mice

**Fig. 3:**
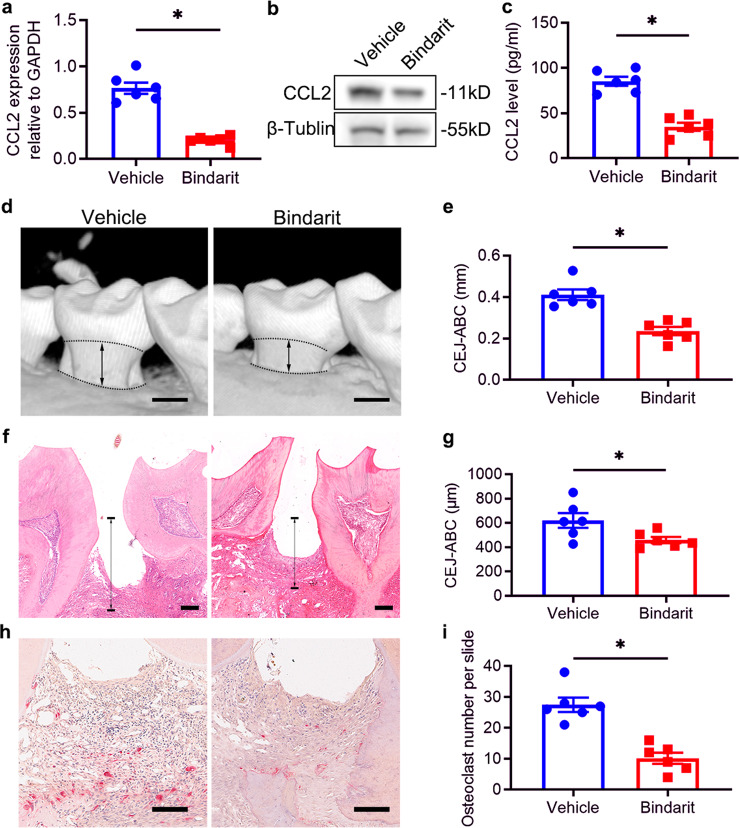
Inhibition of CCL2 production by bindarit rescues epithelial lesions and alveolar bone loss in DP mice. **a** CCL2 levels in gingiva extracted from the periodontium of bindarit-treated and vehicle-treated DP mice were analyzed by RT-qPCR on day 28 after ligature removal (*n* = 6 per group). Error bars indicate SEM. *p < 0.05 (unpaired two-tailed Student’s *t* test). **b** Representative western blots showing the CCL2 levels in the periodontium of each group (*n* = 6 per group) on day 28 after ligature removal. **c** Serum CCL2 levels in each group (*n* = 6 per group) were analyzed by ELISA on day 28 after ligature removal. Error bars indicate SEM. *p < 0.05 (unpaired two-tailed Student’s *t* test). **d** 3D reconstructions of maxillae from each group (*n* = 6 per group) were generated by micro-CT on day 28 after ligature removal. The vertical line extends from the CEJ to the ABC. The CEJ-ABC distance was measured at six predetermined sites on both the buccal and palatal sides. Scale bar = 250 μm. Error bars indicate SEM. **e** Statistical analysis of the CEJ-ABC distance in each group (*n* = 6 per group) as analyzed by micro-CT. Error bars indicate SEM. *p < 0.05 (unpaired two-tailed Student’s *t* test). **f** Histological H&E-stained sections of the periodontium of each group on day 28 after ligature removal. The vertical line extends from the CEJ to the ABC. The CEJ-ABC distance was quantified in each microscopic field of view. Scale bar = 50 μm. **g** Statistical analysis of the CEJ-ABC distance in each group (*n* = 6 per group) as analyzed by H&E staining. Error bars indicate SEM. *p < 0.05 (unpaired two-tailed Student’s *t* test). **h** Histological TRAP-stained sections of the periodontium of each group on day 28 after ligature removal. Osteoclasts are stained red. The number of osteoclasts was quantified in each microscopic field of view. Scale bar = 50 μm. **i** Statistical analysis of the number of osteoclasts in each group (*n* = 6 per group) as determined by TRAP staining. Error bars indicate SEM. *p < 0.05 (unpaired two-tailed Student’s *t* test).

Bindarit downregulates the expression of CCL2, which promotes sustained inflammation through monocyte recruitment.^44^ Thus, we further investigated whether bindarit could be used to treat DP. Bindarit (50 mg/kg) or vehicle was administered to experimental mice daily for 28 days following ligature removal, and specimens were collected from the mice on day 28. RT-qPCR analysis demonstrated that CCL2 mRNA expression was significantly reduced in the periodontal tissues of the bindarit-treated DP (DP + bindarit) group compared with those of the vehicle-treated (DP + vehicle) group (Fig. 3a). Western blot analysis further confirmed the downregulation of CCL2 at the protein level (Fig. 3b). Serum CCL2, which can be secreted by inflamed tissues, contributes to the recruitment of monocytes to inflamed sites, forming a positive feedback loop that enhances the inflammatory response. The ELISA results demonstrated that serum CCL2 levels were decreased in the DP + bindarit group compared with the DP + vehicle group (Fig. 3c).

Subsequently, periodontal tissues were collected from the experimental mice and subjected to micro-CT and histological analysis. Because the inflammatory response can lead to the resorption of alveolar bone and subsequent tooth loss,^45^ we investigated the impact of bindarit treatment on alveolar bone. Micro-CT analysis showed that alveolar bone loss was lower in the DP + bindarit group than in the DP + vehicle group (Fig. 3d, e). In addition, H&E staining revealed that the epithelial layers of periodontal tissues were thicker, the number of infiltrating inflammatory cells was lower, and the amount of neonatal alveolar bone was greater in the DP + bindarit group than in the DP + vehicle group (Fig. 3f, g). Osteoclasts participate in bone resorption and negatively regulate the formation of neonatal alveolar bone. TRAP staining revealed significantly fewer osteoclasts in the periodontal tissues of the DP + bindarit group than in those of the DP + vehicle group (Fig. 3h, i). These results indicate that bindarit administration alleviates DP and rescues alveolar bone loss in DP mice.

### Bindarit administration alleviates periodontal inflammation in DP mice

**Fig. 4:**
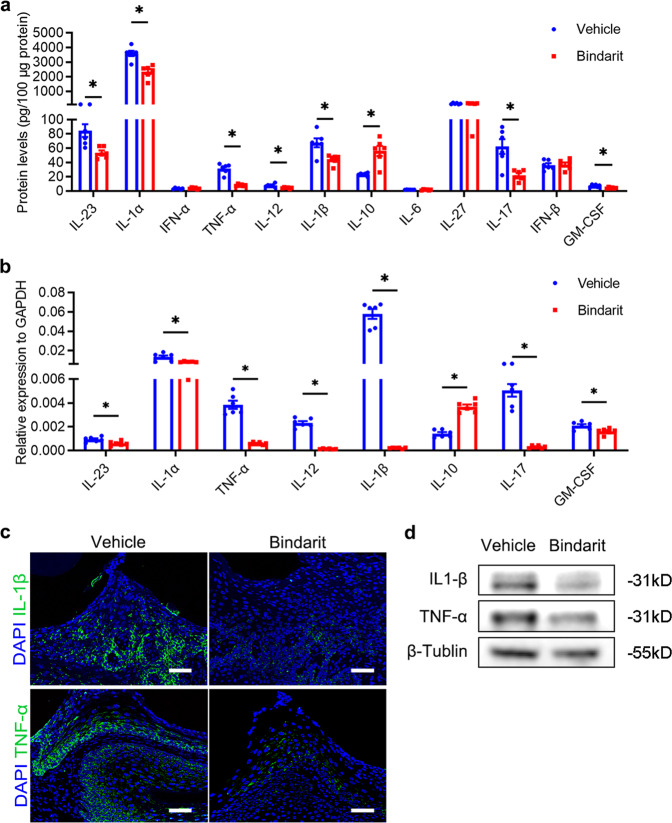
Bindarit administration alleviates periodontal inflammation in DP mice. **a** The levels of various inflammatory cytokines in the periodontium of bindarit-treated and vehicle-treated DP mice on day 28 after ligature removal were analyzed by LEGENDplex^TM^ bead-based immunoassays (*n* = 6 per group). Error bars indicate SEM. *p < 0.05 (unpaired two-tailed Student’s *t* test). **b** The mRNA expression levels of inflammatory cytokines in the periodontium of each group on day 28 after ligature removal were analyzed by RT-qPCR (*n* = 6 per group). Error bars indicate SEM. *p < 0.05 (unpaired two-tailed Student’s *t* test). **c** IL-1β and TNF-α expression levels in the periodontium of each group on day 28 after ligature removal were measured by IF staining. Nuclei were stained with DAPI. Scale bar = 25 μm. **d** IL-1β and TNF-α levels in the periodontium of each group (*n* = 6 per group) on day 28 after ligature removal were measured by western blotting. *p < 0.05 (unpaired two-tailed Student’s *t* test).

Persistently elevated inflammation contributes to the progression of DP.^11,13^ Therefore, we investigated the impact of bindarit on proinflammatory cytokine and chemokine expression in the periodontal tissues of DP mice. The results of LEGENDplex^TM^ bead-based immunoassays showed that the levels of IL-23, IL-1α, TNF-α, IL-12, IL-1β, IL-17, and GM-CSF were reduced, whereas that of the anti-inflammatory cytokine IL-10 was increased in periodontal tissues of DP mice after bindarit administration. Notably, TNF-α, IL-1β, and IL-17 levels decreased by more than 74%, 34%, and 65%, respectively, and the IL-10 level increased by more than 1.3-fold in periodontal tissues of DP + bindarit mice compared with those of DP + vehicle mice (Fig. 4a). RT-qPCR analysis demonstrated that TNF-α, IL-12, IL-1β, and IL-17 mRNA expression was reduced by 84%, 94%, 98%, and 93%, respectively, and that IL-10 mRNA expression was increased by 1.6-fold in periodontal tissues of DP + bindarit mice compared with those of DP + vehicle mice (Fig. 4b). Western blot analysis and IF staining further confirmed the downregulation of IL-1β and TNF-α at the protein level in the DP + bindarit group compared with the DP + vehicle group (Fig. 4c, d and Supplementary Fig. 2c). Taken together, these data suggest that bindarit can reduce periodontal inflammation in DP mice.

### Bindarit administration suppresses infiltration of the periodontium of DP mice by proinflammatory monocytes

**Fig. 5:**
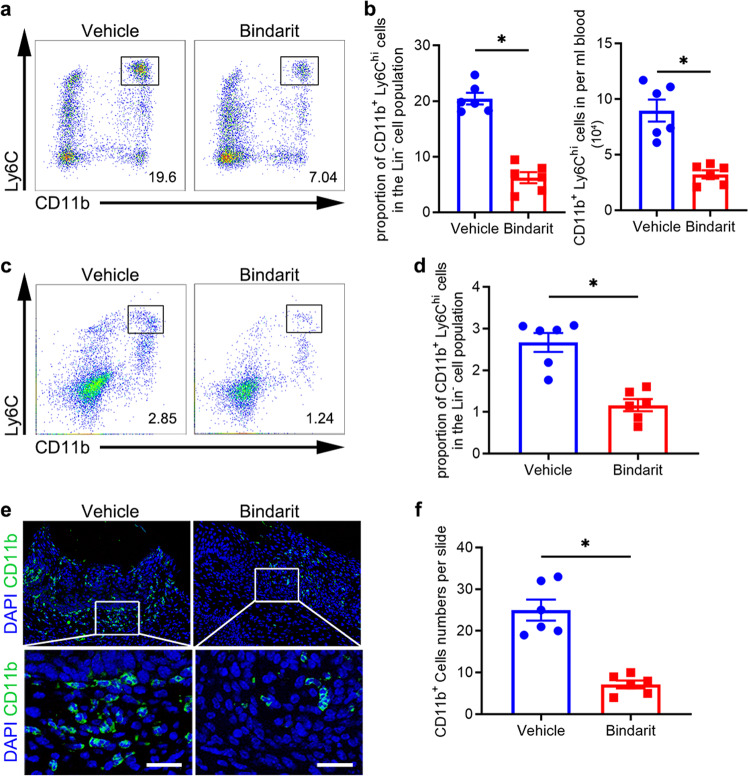
Bindarit administration suppresses infiltration of the periodontium of DP mice by proinflammatory monocytes. **a** The proportion of CD11b^+^Ly6C^hi^ cells in the Lin^-^ cell population in blood from bindarit-treated and vehicle-treated DP mice on day 28 after ligature removal was analyzed by flow cytometry. Gates captured single, live Lin^–^ cells in blood (*n* = 6 per group). **b** Statistical analysis of flow cytometry data describing the proportion and quantity of CD11b^+^Ly6C^hi^ cells in an individual, live Lin^-^ cell population in blood from each group (*n* = 6 per group). Error bars indicate SEM. *p < 0.05 (unpaired two-tailed Student’s *t* test). **c** The proportion of CD11b^+^Ly6C^hi^ cells in an individual, live Lin^-^ cell population in the periodontium of each group (*n* = 6 per group) was analyzed by flow cytometry on day 28 after ligature removal. Gates captured individual, live Lin^–^ cells in the periodontium. **d** Statistical analysis of flow cytometry data describing the proportion of periodontal CD11b^+^Ly6C^hi^ cells in an individual, live Lin^-^ cell population in each group (*n* = 6 per group). Error bars indicate SEM. *p < 0.05 (unpaired two-tailed Student’s *t* test). **e** CD11b^+^ cells in the periodontium of each group on day 28 after ligature removal were analyzed by IF staining. Nuclei were stained with DAPI. Scale bar = 25 μm. **f** The number of CD11b^+^ cells in each microscopic field of view was quantified. Statistical analysis of the IF staining of periodontal CD11b^+^ cells in each group (*n* = 6 per group) is shown. Error bars indicate SEM. *p < 0.05 (unpaired two-tailed Student’s *t* test).

Proinflammatory monocytes cause persistent inflammation by producing inflammatory cytokines and tissue-degrading enzymes.^16,46^ Hence, we investigated whether bindarit alleviated DP by reducing proinflammatory monocyte infiltration. Flow cytometric analysis showed that the proportion and quantity of CD11b^+^Ly6C^hi^ cells in peripheral blood were significantly reduced in the DP + bindarit group compared with the DP + vehicle group (Fig. 5a, b; Supplementary Fig. 6). We further found that the proportion of CD11b^+^Ly6C^hi^ cells in periodontal tissues was significantly reduced in the DP + bindarit group compared with the DP + vehicle group (Fig. 5c, d; Supplementary Fig. 4). Moreover, IF staining analysis revealed fewer infiltrating CD11b^+^ cells in the DP + bindarit group than in the DP + vehicle group (Fig. 5e, f and Supplementary Fig. 2d). Since CD4^+^ T cells participate in periodontitis-induced bone loss, we next investigated the impact of bindarit on the recruitment of CD4^+^ T cells to the inflamed periodontium. IF staining revealed that the number of CD4^+^ T cells in the periodontium was not affected by 7 days of bindarit treatment. In contrast, the number of proinflammatory monocytes in the periodontium decreased by approximately 52% after 7 days of bindarit treatment (Supplementary Fig. 7 and Supplementary Fig. 2e). These findings indicate that bindarit suppresses periodontal inflammation in DP mice mainly by reducing the infiltration of periodontal tissues by proinflammatory monocytes.

### Bindarit administration alters the inflammatory properties of macrophages in the periodontium of DP mice

**Fig. 6:**
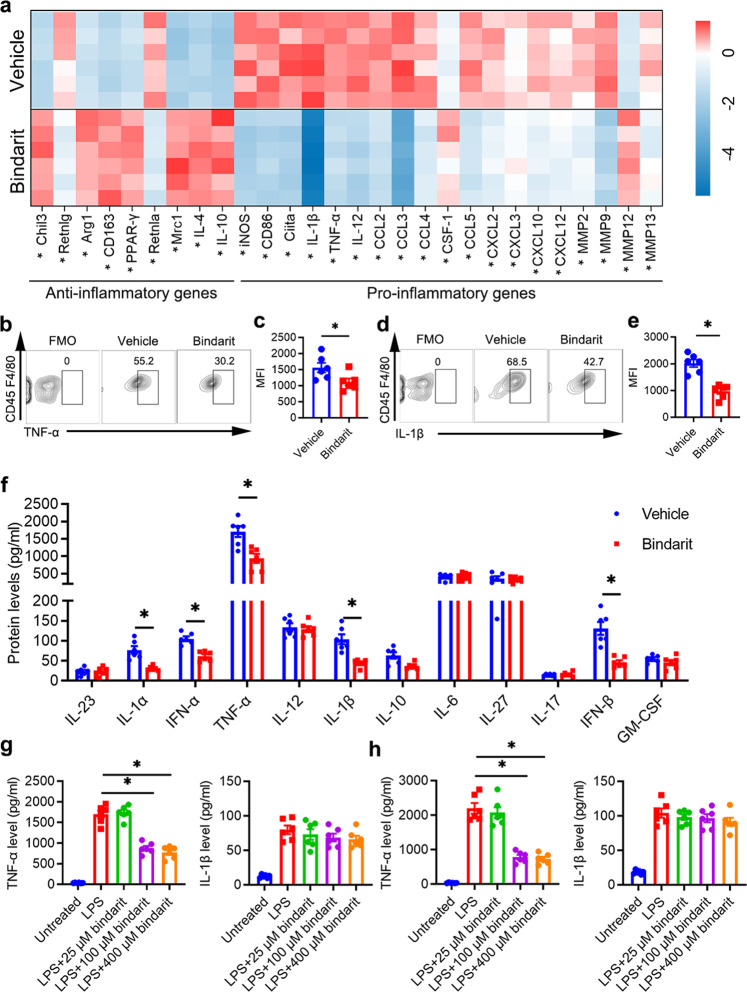
Bindarit administration alters the inflammatory properties of macrophages in the periodontium of DP mice. **a** The mRNA expression levels of various cytokines and chemokines in CD45^+^F4/80^+^ cells isolated from the periodontium of mice in the DP + bindarit and DP + vehicle groups on day 28 after ligature removal were analyzed by RT-qPCR (*n* = 6 per group). *p < 0.05 (unpaired two-tailed Student’s *t* test). **b**, **d** The percentages of TNF-α and IL-1β with the fluorescence minus one (FMO) control in CD45^+^F4/80^+^ cells isolated from the periodontium of each group were analyzed by flow cytometry. **c**, **e** Statistical analysis of the flow cytometry data describing the MFI of TNF-α, and IL-1β with the FMO control in the CD45^+^F4/80^+^ cell population of each group (*n* = 6 per group). Error bars indicate SEM. *p < 0.05 (unpaired two-tailed Student’s *t* test). **f** The levels of various cytokines in the culture supernatant of CD45^+^F4/80^+^ cells from each group on day 28 after ligature removal were analyzed using LEGENDplex^TM^ bead-based immunoassays (*n* = 6 per group). Error bars indicate SEM. *p < 0.05 (unpaired two-tailed Student’s *t* test). **g** The levels of TNF-α and IL-1β in isolated macrophages from *db/db* mice after bindarit pretreatment and LPS stimulation were analyzed by ELISA (*n* = 6 per group). Error bars indicate SEM. *p < 0.05 (one-way ANOVA). **h** The levels of TNF-α and IL-1β in isolated blood monocytes from *db/db* mice after bindarit pretreatment and LPS stimulation were analyzed by ELISA (*n* = 6 per group). Error bars indicate SEM. *p < 0.05 (one-way ANOVA).

Macrophages undergo phenotypic and functional changes during the stages of tissue repair.^41^ Macrophages can be classified as proinflammatory macrophages and anti-inflammatory macrophages.^47^ Proinflammatory macrophages release multiple inflammatory cytokines, thereby participating in the progression of inflammation.^47,48^ In contrast, anti-inflammatory macrophages are involved in the resolution of inflammation.^47,48^ RT-qPCR analysis showed that the mRNA levels of genes related to the proinflammatory phenotype, including inducible nitric oxide synthase (iNOS) and CD86, were significantly decreased in macrophages isolated from the DP + bindarit group compared with macrophages isolated from the DP + vehicle group. Additionally, genes related to the anti-inflammatory phenotype, including the genes encoding chitinase-like protein 3 (Chil3), arginase (Arg1), macrophage mannose receptor 1 (Mrc1), IL-4 and IL-10, were upregulated in macrophages isolated from the DP + bindarit group compared with those isolated from the DP + vehicle group. Moreover, macrophages isolated from the DP + bindarit group exhibited decreased expression of proinflammatory chemokines and cytokines and extracellular matrix-modulating proteins, including IL-1β, TNF-α, CCL2, CCL5, MMP2, and MMP9 (Fig. 6a). These results indicate that more anti-inflammatory macrophages were generated in the DP + bindarit group than in the DP + vehicle group.

Because the cytokines released from macrophages can indicate the properties of the macrophages,^49^ we further evaluated inflammatory cytokine expression in isolated macrophages. Earlier in this work, we showed that TNF-α and IL-1β were significantly downregulated in DP + bindarit mice compared with DP + vehicle mice (Fig. 4). Here, we further found that macrophages isolated from DP + bindarit mice produced lower levels of TNF-α and IL-1β than macrophages isolated from DP + vehicle mice, as evidenced by flow cytometric analysis after ex vivo stimulation (Fig. 6b–e). Isolated macrophages were then cultured and stimulated with 100 ng/ml LPS for 24 h. The results of LEGENDplex^TM^ bead-based immunoassays indicated that the levels of IL-1α, IFN-α, TNF-α, IL-1β, and IFN-β were decreased by more than 60%, 42%, 45%, 58%, and 65%, respectively, in the supernatant of macrophages from DP + bindarit mice compared with those from DP + vehicle mice (Fig. 6f). Taken together, these data indicate that bindarit can diminish the proinflammatory properties of macrophages in the periodontal tissues of DP mice.

### Bindarit treatment partially blocks LPS-induced cytokine release from macrophages and monocytes

The efficacy of bindarit in DP could involve suppression of proinflammatory monocyte infiltration and alterations in the inflammatory properties of macrophages. To investigate the direct anti-inflammatory effects of bindarit on periodontal macrophages and blood monocytes, we measured cytokine secretion by macrophages and monocytes isolated from DP mice after pretreatment with bindarit and stimulation with LPS. LPS stimulation caused significant release of TNF-α and IL-1β by macrophages and monocytes. LPS-induced TNF-α release from macrophages was not affected by treatment of the cells with 25 μM bindarit but was suppressed by more than 62% in cells treated with 100 μM or 400 μM bindarit (Fig. 6g). Similarly, 100 μM and 400 μM bindarit suppressed TNF-α release from monocytes by more than 60% (Fig. 6h). However, bindarit did not affect LPS-induced IL-1β release from macrophages and monocytes (Fig. 6g, h). These results suggest that bindarit partially blocks LPS-induced cytokine release from macrophages and monocytes.

## Discussion

Persistently elevated inflammation contributes to DP. In this study, we found for the first time that CCL2 inhibition by bindarit administration reduced alveolar bone loss and increased periodontal epithelial thickness by alleviating periodontal inflammation. Specifically, bindarit alleviated periodontal inflammation by suppressing excessive proinflammatory monocyte infiltration and altering the inflammatory properties of macrophages.

Due to their involvement in immune cell trafficking, chemokines participate in the progression of multiple inflammatory diseases.^50^ CCL2 levels are persistently elevated in the gingiva of patients with periodontitis, and this increase is reported to be associated with persistent periodontal inflammation.^25^ However, the mechanism through which CCL2 contributes to the development of DP remains unclear. Hence, we induced DP in mice to mimic the state of persistent CCL2 elevation in patients with DP. GO enrichment analysis showed that the upregulated DEGs in DP mice were associated with CCR2 chemokine receptor binding. The CCR2 ligands CCL2, CCL7, and CCL12 were highly upregulated in DP mice, and the expression of CCL2 was the highest among them. CCL2 has been reported to be more effective than CCL7 or CCL12 in recruiting proinflammatory monocytes to injured sites.^51^ We showed that CCL2 levels were persistent in DP mice, whereas they rapidly decreased in NP mice within 9 days after ligature removal. Thus, we concluded that persistent CCL2 elevation accelerates the progression of DP by recruiting proinflammatory monocytes to inflamed periodontal tissues.

Bindarit, a CCL2 inhibitor that has been proven to be effective in the treatment of various inflammatory diseases in mouse models through its ability to suppress proinflammatory monocyte infiltration,^22,52^ is a promising drug for the treatment of DP. Thus, we investigated the impact of bindarit on DP. Alveolar bone loss and epithelial damage are the prominent features of periodontitis. We found that bindarit reduced alveolar bone loss and increased the thickness of the periodontal epithelium in DP mice. The periodontal epithelium is a physical barrier that provides a defense against pathogen invasion. Recovery of the periodontal periodontium to a healthy state could protect periodontal tissues from pathogen invasion. As persistent inflammation contributes to excessive damage to alveolar bone and the periodontal epithelium, we measured the impact of bindarit on the inflammatory state of the periodontium. We found that the levels of various inflammatory cytokines, especially TNF-α, were markedly reduced in the bindarit-treated group compared to the vehicle-treated group. Notably, diabetes-enhanced TNF-α has been shown to be important in the progression of DP by prolonging inflammation and promoting osteoclastogenesis in the periodontium.^28^ Inhibition of TNF-α by blockade has been found to reduce alveolar bone resorption in rat models of DP.^12^ Hence, one of the important mechanisms through which bindarit reduced the number of osteoclasts and alveolar bone loss in DP mice is the reduction of TNF-α levels in the periodontium. In addition, proinflammatory cytokines have been reported to aggravate periodontitis by inducing NF-κB activation in osteoblasts, a process that plays an important role in bone resorption.^53^ We deduced that bindarit may reduce NF-κB activation in osteoblasts by suppressing periodontal inflammation, thereby reducing alveolar bone loss.

Macrophages play a critical role in mediating periodontal inflammation.^13,43^ The balance between proinflammatory and anti-inflammatory macrophages is critical in the resolution of tissue inflammation.^13,48^ A high ratio of proinflammatory macrophages to anti-inflammatory macrophages leads to aberrant repair of the periodontium.^13,42^ Interestingly, we found that various genes related to the proinflammatory phenotype, including iNOS, CD86, IL-1β, and TNF-α, were significantly downregulated and that genes related to the anti-inflammatory phenotype, such as Chil3, Arg1, Mrc1, IL-4, and IL-10, were upregulated in macrophages in the periodontal tissues of the bindarit-treated group compared with those of the vehicle-treated group. These results indicated that more anti-inflammatory macrophages were generated after bindarit treatment. Moreover, the levels of inflammatory cytokines, including TNF-α and IL-1β, in isolated periodontal macrophages were significantly lower in the bindarit-treated group than in the vehicle-treated group. Reducing the levels of proinflammatory cytokines derived from macrophages has been proven to be effective in suppressing periodontal inflammation and alveolar bone resorption.^42,43^ Our results indicated that bindarit decreased the production of proinflammatory cytokines by macrophages in the periodontium, thereby alleviating periodontitis. MMP2 and MMP9 cause periodontal destruction by degrading type I collagen, the main component of the extracellular matrix of the periodontium.^18^ The gene expression levels of MMP2 and MMP9 in periodontal macrophages were significantly downregulated in the bindarit-treated group compared with the vehicle-treated group. All of these results indicate that bindarit reduced the levels of inflammatory cytokines and MMPs produced by macrophages in the inflamed periodontium under diabetic conditions, thus improving the periodontal microenvironment in DP.

T cells, especially CD4^+^ T cells, contribute to periodontitis by secreting inflammatory mediators such as TNF-α.^54^ CCL2 has been reported to be involved in the infiltration of inflamed sites by CD4^+^ T cells.^55^ Thus, we investigated the impact of bindarit on the recruitment of CD4^+^ T cells to the inflamed periodontium. We showed that the number of CD4^+^ T cells in the periodontium was not significantly affected in mice treated with bindarit. We deduced that other chemokines such as CCL5, CCL20, and CXCL9 might be involved in CD4^+^ T cell recruitment to the inflamed periodontium.^20^ However, the identification of other subsets of CD4^+^ T cells that might be affected by bindarit will require further study. Despite its limited impact on CD4^+^ T-cell recruitment, bindarit alleviated DP. Based on the fact that bindarit was found to suppress infiltration of the periodontium by proinflammatory monocytes that aggravate periodontitis, we concluded that bindarit suppresses periodontal inflammation mainly by reducing proinflammatory monocyte recruitment.

In addition to its chemotactic effects on immune cells, CCL2 probably plays additional roles in the progression of inflammation-related diseases. CCL2 has been reported to mediate the polarization of macrophages that participate in tissue inflammation. For example, Zhuang et al. reported that CCL2 caused bone marrow-derived macrophages (BMDMs) to polarize toward the M2 phenotype (anti-inflammatory phenotype).^42^ However, Danh et al. showed that CCl2 induced BMDMs to polarize to the M1 phenotype (proinflammatory phenotype) through the CCR2/RhoA axis.^56^ The impact of CCL2 on macrophage polarization thus appears inconsistent. Accumulating evidence indicates that CCL2 aggravates inflammation through other pathways. CCL2 has been reported to induce the expression of genes related to inflammation by binding to CCR2. For example, CCL2 causes upregulation of MCP-1-induced protein (MCPIP) in cardiomyocytes in a mouse model of heart failure.^57^ MCPIP induces cardiomyocyte apoptosis by causing ROS accumulation and endoplasmic reticulum stress in cardiomyocytes.^57^ Osteoclasts participate in inflammation-induced bone resorption. CCL2 has been reported to promote the differentiation of monocytes to preosteoclasts by inducing MCPIP in monocytes.^58^ CCL2 and CCL7 have also been shown to enhance RANK levels in preosteoclasts via the NF-κB and ERK1/2 signaling pathways.^31^ RANKL further promotes the differentiation of preosteoclasts into osteoclasts.^32^ In this study, the upregulated CCL2 and CCL7 levels observed in the periodontium of DP mice may promote osteoclast differentiation by increasing RANK levels in preosteoclasts, thereby causing alveolar bone resorption. Similarly, the protective effects of bindarit on reducing osteoclast numbers in the DP-affected periodontium may have involved the suppression of RANK expression on the surface of preosteoclasts. However, the possibility that CCL2 plays additional roles in the progression of DP needs further investigation.

In conclusion, this study demonstrated that the inhibition of CCL2 production by the administration of bindarit alleviated DP by suppressing infiltration of the periodontium by proinflammatory monocytes and altering the inflammatory properties of macrophages. This study provides a novel and effective therapeutic approach for DP.

## Supplementary information

Supplementary materials
