# Supplementary material for: Inhibition of CCL2 by bindarit alleviates diabetes-associated periodontitis by suppressing inflammatory monocyte infiltration and altering macrophage properties
Source: Cell Mol Immunol. 2020 Jul 16;18(9):2224–35. doi: 10.1038/s41423-020-0500-1 (PMC8429574; doi:10.1038/s41423-020-0500-1)
Supplement: Supplementary file 1 — Supplementary materials [file 41423_2020_500_MOESM1_ESM.docx]

**Supplementary Materials**

**Inhibition of CCL2 by bindarit alleviates** **diabetes-associated periodontitis by suppressing inflammatory monocyte infiltration and altering macrophage properties**

Zongshan Shen^1*^, Shuhong Kuang^1*^, Min Zhang^2,3*^, Xin Huang^1^, Jiayao Chen^1^, Meiliang Guan^1^, Wei Qin^1,4#^, Hockin H. K. Xu^4#^, Zhengmei Lin^1#^

1. Hospital of Stomatology, Guangdong Provincial Key Laboratory of Stomatology, Guanghua School of Stomatology, Sun Yat-sen University, Guangzhou, Guangdong, China

2. Department of Andrology, The First Affiliated Hospital, Sun Yat-sen University, Guangzhou, Guangdong, China

3. The Key Laboratory for Stem Cells and Tissue Engineering, Center for Stem Cell Biology and Tissue Engineering, Ministry of Education, Sun Yat-sen University, Guangzhou, Guangdong, China

4. Department of Advanced Oral Sciences & Therapeutics, University of Maryland School of Dentistry, Baltimore, MD, USA; Center for Stem Cell Biology and Regenerative Medicine, University of Maryland School of Medicine, Baltimore, MD, USA; University of Maryland Marlene and Stewart Greenebaum Cancer Center, University of Maryland School of Medicine, Baltimore, MD, USA

^*^These three authors contributed equally to this work.

^#^Corresponding authors:

Zhengmei Lin; Address: 56 Ling-yuan West Road, Guangzhou, China,

Tel: +86-20-87335986, Fax: +86 20 83822807, E-mail: [linzhm@mail.sysu.edu.cn](mailto:linzhm@mail.sysu.edu.cn)

Hockin H. K. Xu; Address: 650 West Baltimore Street, Baltimore, MD 21201, USA

Tel: 443-562-1295, Fax: 410-706-1565, E-mail: hxu@umaryland.edu

Wei Qin; Address: 56 Ling-yuan West Road, Guangzhou, China,

Tel: +86-20-83861544, Fax: +86-20-83822807, E-mail: qinwei2@mail.sysu.edu.cn


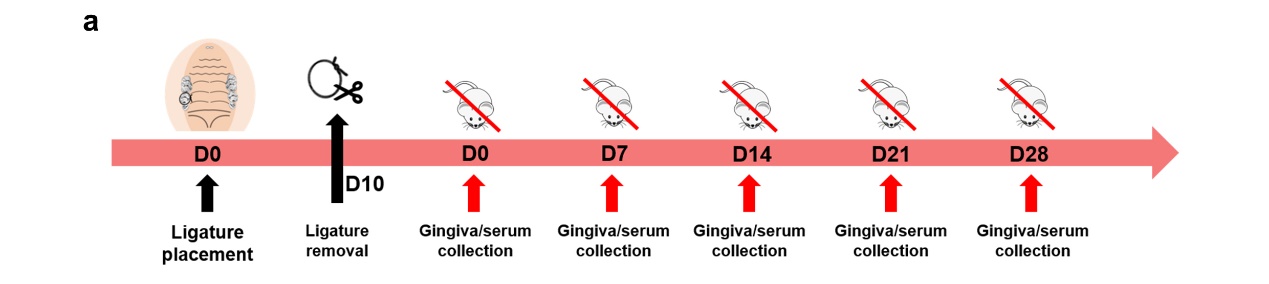


**Figure S1. Experimental protocol**

(a) Serum CCL2 levels and the proportion of CD11b^+^Ly6C^hi^ cells in the periodontium of DP and D mice were determined on days 0, 7, 14, 21 and 28 after ligature removal.


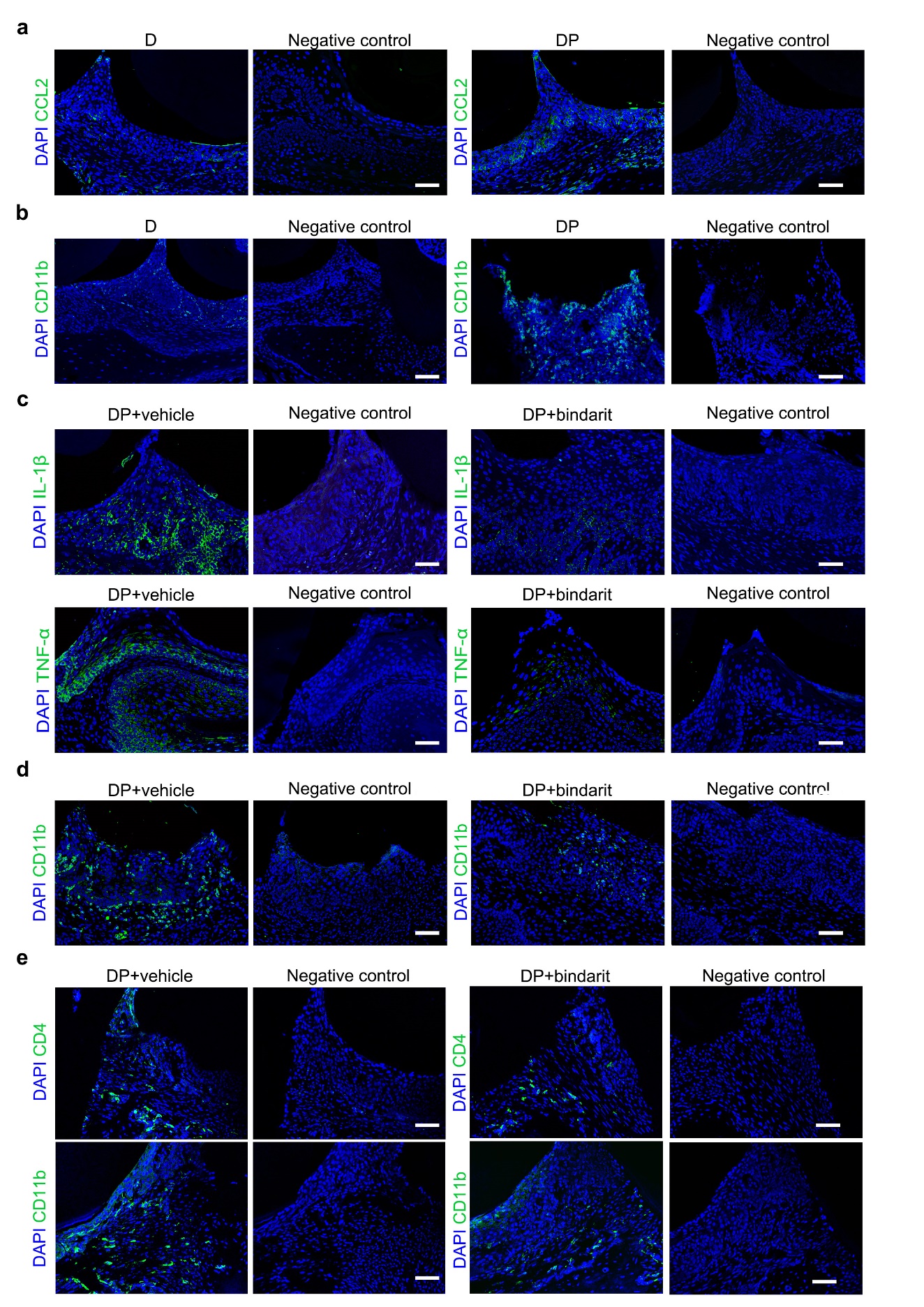


**Figure S2. Negative controls for the immunofluorescence analysis**

1. IF staining and negative control for CCL2 expression in the periodontium of D and DP mice on day 10 after ligature placement. Nuclei were visualized with DAPI staining (blue). Scale bar = 100 μm.
2. IF staining and negative control for CD11b^+^ cells in the periodontium of D and DP mice on day 28 after ligature removal. Nuclei were visualized with DAPI staining (blue). Scale bar = 100 μm.
3. IF staining and negative control for IL-1β and TNF-α expression in the periodontium of bindarit-treated and vehicle-treated DP mice on day 28 after ligature removal. Nuclei were visualized with DAPI staining (blue). Scale bar = 100 μm.
4. IF staining and negative control for CD11b^+^ cells in the periodontium of bindarit-treated and vehicle-treated DP mice on day 28 after ligature removal. Nuclei were visualized with DAPI staining (blue). Scale bar = 100 μm.
5. IF staining and negative control for CD4^+^ and CD11b^+^ cells in the periodontium of bindarit-treated and vehicle-treated DP mice on day 7 after ligature removal. Nuclei were visualized with DAPI staining (blue). Scale bar = 100 μm.


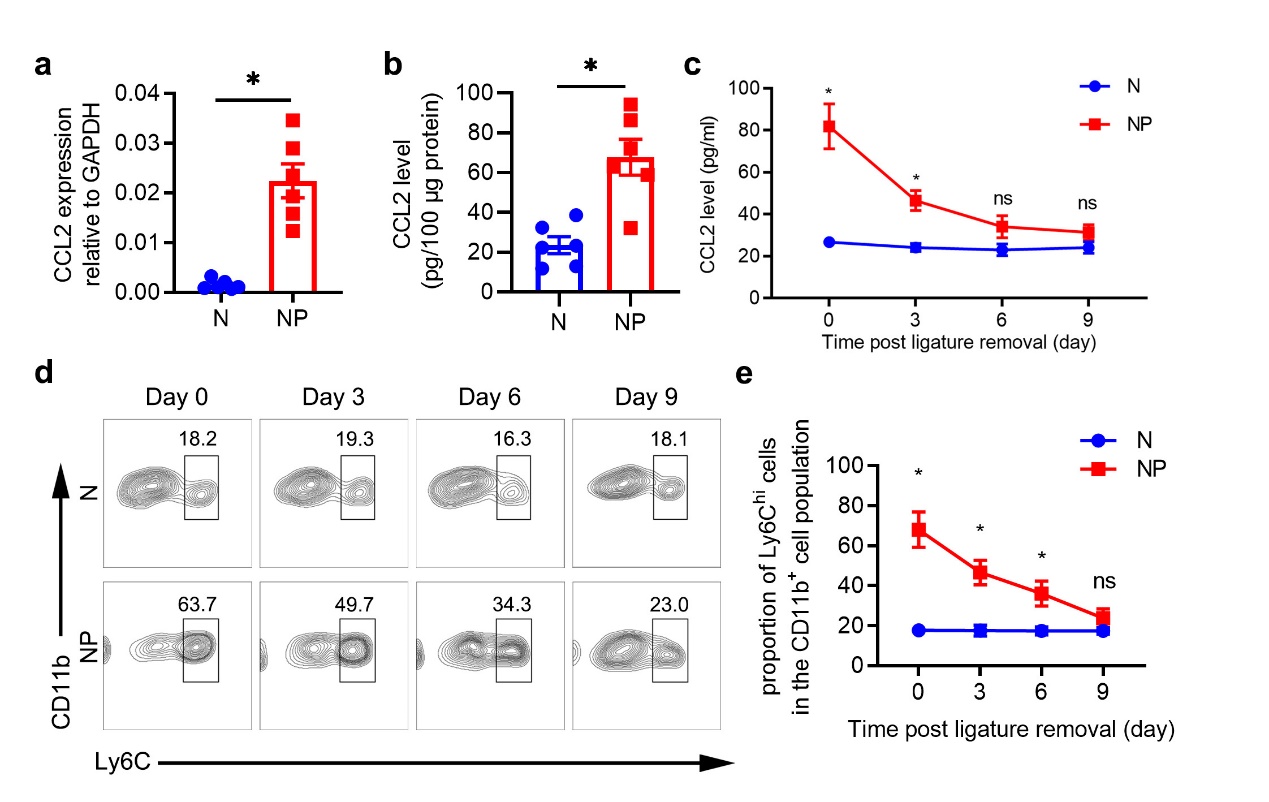


**Figure S3. CCL2 expression and proinflammatory monocyte infiltration in NP mice and N mice**

1. The CCL2 mRNA expression levels in the periodontium of NP mice (n=6) and N mice (n=6) at 10 days after ligature placement were analysed by RT-qPCR. Error bars indicate SEM. **p* < 0.05 (unpaired two-tailed Student’s *t* test).
2. The CCL2 levels in cells extracted from the periodontium of each group (n = 6 per group) were analysed by ELISA 10 days after ligature placement. Error bars indicate SEM. **p* < 0.05 (unpaired two-tailed Student’s *t* test).
3. The serum levels of CCL2 in each group were analysed by ELISA on days 0, 3, 6 and 9 after ligature removal (ligature-induced periodontitis mode, n = 6 per group at the indicated time points). Error bars indicate SEM. **p* < 0.05 (unpaired two-tailed Student’s *t* test).
4. The proportion of Ly6C^hi^ cells in the CD11b^+^ cell population in the periodontium of NP mice and N mice was analysed by flow cytometry on days 0, 3, 6 and 9 after ligature removal (n = 6 per group at each time point). Gates captured single, live Lin [CD3, CD19, NK1.1, Ly6G]^–^CD11b^+^ cells in the periodontium.
5. Statistical analysis of flow cytometry data showing the proportion of periodontal Ly6C^hi^ cells in the single, live Lin^-^CD11b^+^ cell population of each group (n = 6 per group at each time point). Error bars indicate SEM. **p* < 0.05 (unpaired two-tailed Student’s *t* test).


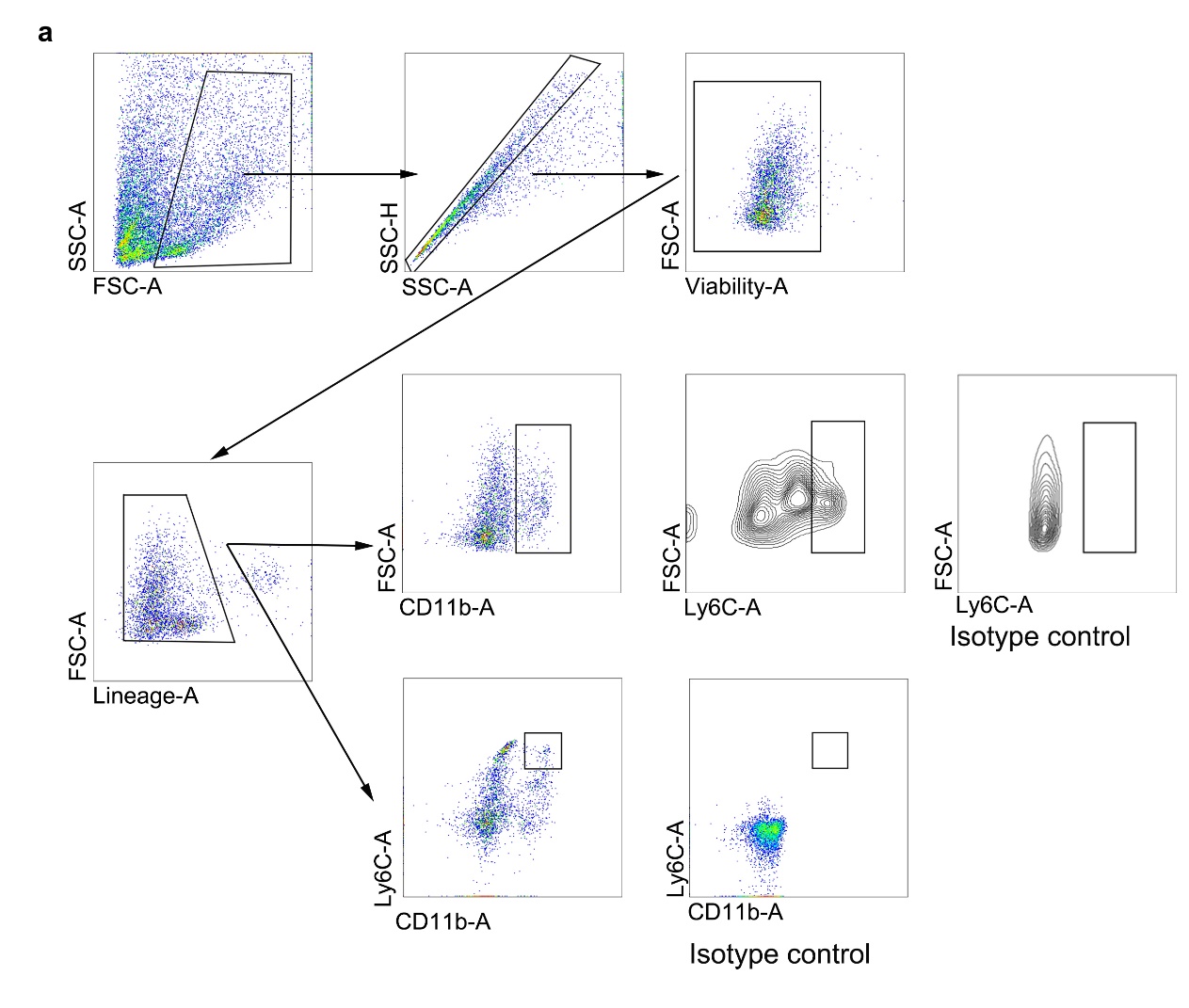


**Figure S4. Gating strategies for the flow cytometry analysis of periodontal cells**

(a) Gating strategy used to select single, live CD11b^+^Ly6C^hi^ cells in the CD11b^+^ cell population and live CD11b^+^Ly6C^hi^ cells in the Lin [CD3, CD19, NK1.1, Ly6G]^-^ cell population in the periodontium of experimental mice and the isotype control.


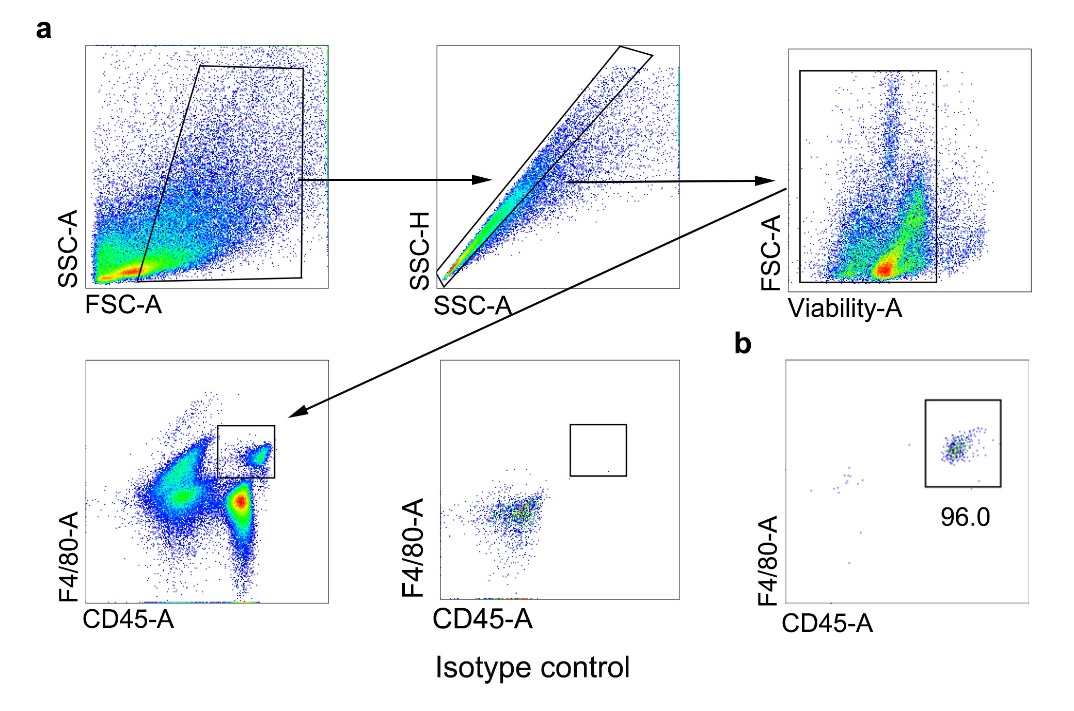


**Figure S5. Gating strategies for FACS of periodontal tissue samples**

(a) Gating strategy used to select single, live CD45^+^F4/80^+^ cells in the periodontium of experimental mice by FACS using an isotype control.

(b) The purity of the sorted CD45^+^F4/80^+^ cells was measured by flow cytometry.


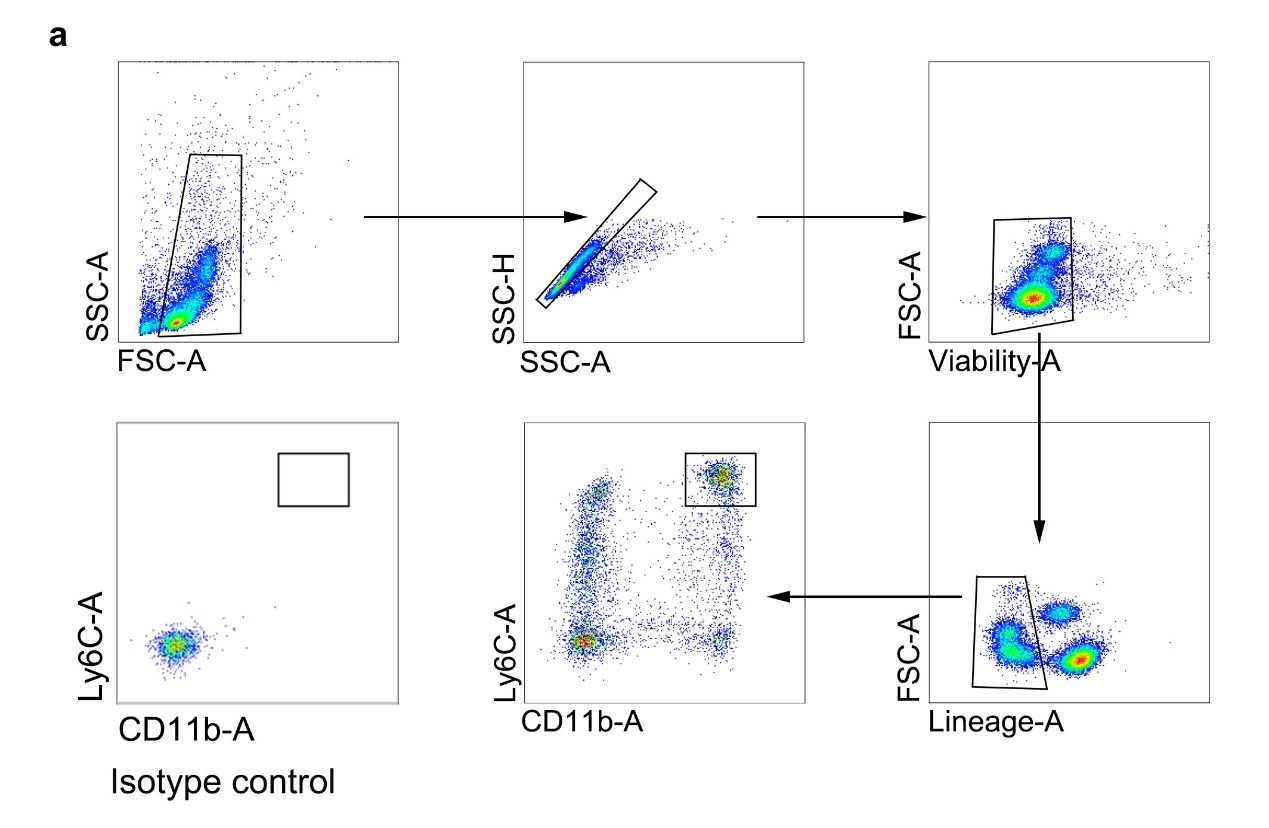


**Figure S6. Gating strategies for the flow cytometry analysis of blood cells**

(a) Gating strategy used to select single, live CD11b^+^Ly6C^hi^ cells in the Lin^-^ cell population in the blood of experimental mice and the isotype control.


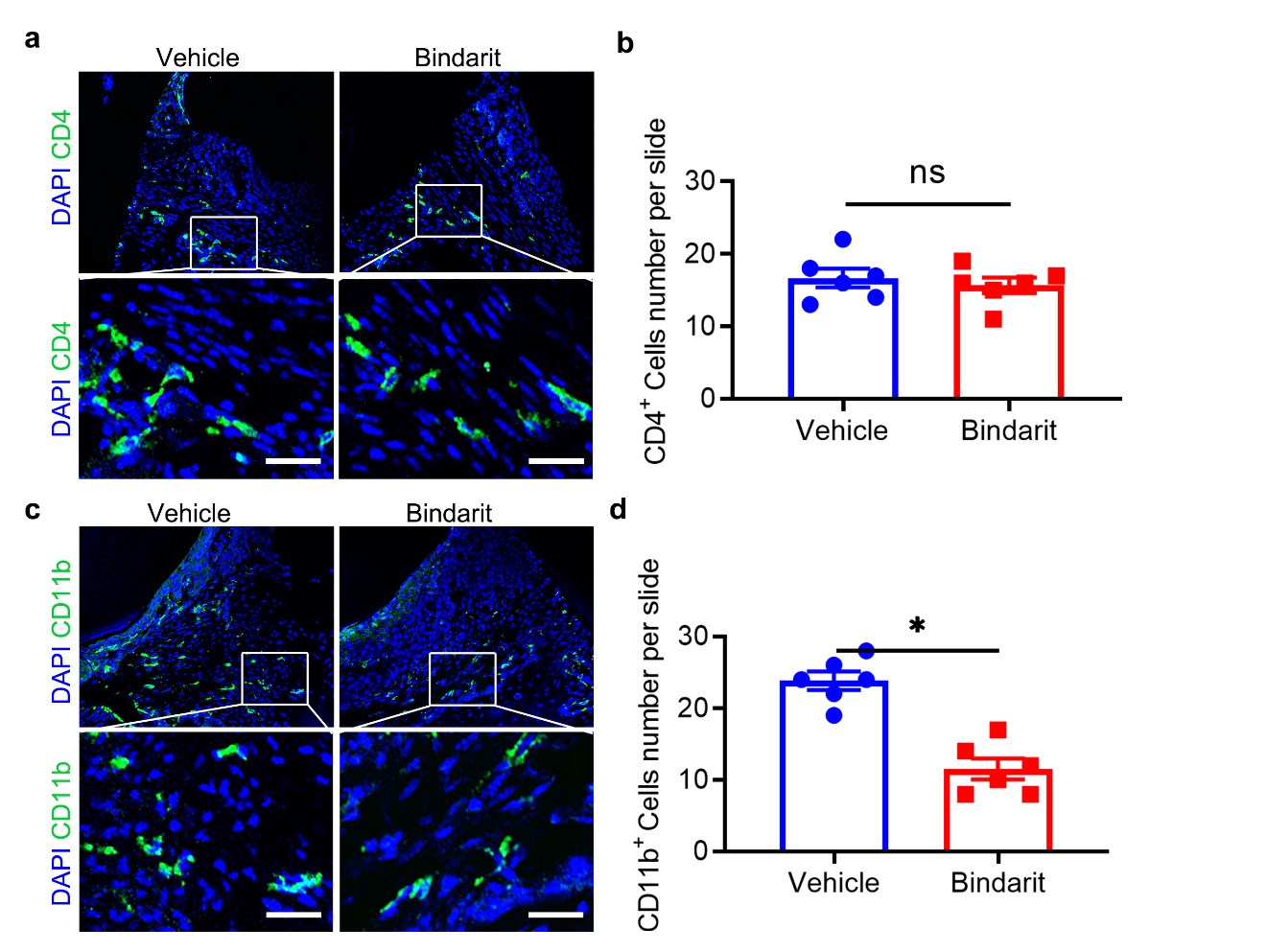


**Figure S7. CD4^+^ T cells and monocytes in the periodontium of mice after 7 days of bindarit treatment**

1. CD4^+^ cells in the periodontium of bindarit-treated and vehicle-treated DP mice on day 7 after ligature removal were detected by IF staining. Nuclei were stained with DAPI. Scale bar = 25 μm.
2. The number of CD4^+^ cells in each microscopic field of view was quantified. Statistical analysis of the IF staining of periodontal CD4^+^ cells in each group (n = 6 per group) is shown. Error bars indicate SEM. **p* < 0.05 (unpaired two-tailed Student’s *t* test).
3. CD11b^+^ cells in the periodontium of each group on day 7 after ligature removal were detected by IF staining. Nuclei were stained with DAPI. Scale bar = 25 μm.
4. The number of CD11b^+^ cells in each microscopic field of view was quantified. Statistical analysis of the IF staining of periodontal CD11b^+^ cells in each group (n = 6 per group) is shown. Error bars indicate SEM. **p* < 0.05 (unpaired two-tailed Student’s *t* test).

**Table S1. DAVID enrichment table**

| Category | Term | P value | Fold Enrich-ment | Bonferroni | FDR |
| --- | --- | --- | --- | --- | --- |
| GOTERM_MF_DIRECT | GO:0005125~cytokine activity | 2.53E-12 | 11.087178 | 4.94E-10 | 3.16E-09 |
| GOTERM_MF_DIRECT | GO:0008009~chemokine activity | 1.54E-09 | 26.169 | 3.01E-07 | 1.93E-06 |
| GOTERM_MF_DIRECT | GO:0030414~peptidase inhibitor activity | 2.30E-07 | 11.346992 | 4.49E-05 | 2.87E-04 |
| GOTERM_MF_DIRECT | GO:0050786~RAGE receptor binding | 1.91E-05 | 69.784 | 0.003722 | 0.023866 |
| GOTERM_MF_DIRECT | GO:0004867~serine-type endopeptidase inhibitor activity | 2.78E-05 | 9.0775935 | 0.005398 | 0.034639 |
| GOTERM_MF_DIRECT | GO:0031727~CCR2 chemokine receptor binding | 2.98E-04 | 104.676 | 0.056441 | 0.371188 |
| GOTERM_MF_DIRECT | GO:0035662~Toll-like receptor 4 binding | 4.94E-04 | 83.7408 | 0.091887 | 0.615073 |
| GOTERM_MF_DIRECT | GO:0042056~chemoattractant activity | 7.19E-04 | 22.33088 | 0.130862 | 0.893749 |
| GOTERM_MF_DIRECT | GO:0004982~N-formyl peptide receptor activity | 0.0010281 | 59.814857 | 0.181749 | 1.275746 |
| GOTERM_MF_DIRECT | GO:0050544~arachidonic acid binding | 0.0017461 | 46.522667 | 0.288795 | 2.157809 |
| GOTERM_MF_DIRECT | GO:0004871~signal transducer activity | 0.0022382 | 2.7999753 | 0.353992 | 2.758134 |
| GOTERM_MF_DIRECT | GO:0004875~complement receptor activity | 0.002643 | 38.064 | 0.403136 | 3.249381 |
| GOTERM_MF_DIRECT | GO:0045236~CXCR chemokine receptor binding | 0.002643 | 38.064 | 0.403136 | 3.249381 |
| GOTERM_MF_DIRECT | GO:0004869~cysteine-type endopeptidase inhibitor activity | 0.0040005 | 12.406044 | 0.542356 | 4.88033 |
| GOTERM_MF_DIRECT | GO:0008201~heparin binding | 0.0044856 | 5.5457483 | 0.583832 | 5.457013 |
| GOTERM_MF_DIRECT | GO:0001664~G-protein coupled receptor binding | 0.0106548 | 8.723 | 0.876168 | 12.51531 |
| GOTERM_MF_DIRECT | GO:0004222~metalloendopeptidase activity | 0.0115326 | 5.6734959 | 0.895851 | 13.47932 |
| GOTERM_MF_DIRECT | GO:0031729~CCR4 chemokine receptor binding | 0.0141652 | 139.568 | 0.938083 | 16.31194 |
| GOTERM_MF_DIRECT | GO:0031726~CCR1 chemokine receptor binding | 0.0141652 | 139.568 | 0.938083 | 16.31194 |
| GOTERM_MF_DIRECT | GO:0003924~GTPase activity | 0.0168098 | 4.0067368 | 0.963329 | 19.07141 |
| GOTERM_MF_DIRECT | GO:0005198~structural molecule activity | 0.0267291 | 3.548339 | 0.994923 | 28.69276 |
| GOTERM_MF_DIRECT | GO:0008237~metallopeptidase activity | 0.0273902 | 4.3615 | 0.995553 | 29.29494 |
| GOTERM_MF_DIRECT | GO:0008233~peptidase activity | 0.0309954 | 2.4343256 | 0.997845 | 32.49758 |
| GOTERM_MF_DIRECT | GO:0004872~receptor activity | 0.0344031 | 4.0572093 | 0.998916 | 35.40151 |
| GOTERM_MF_DIRECT | GO:0048248~CXCR3 chemokine receptor binding | 0.0350406 | 55.8272 | 0.999047 | 35.93186 |
| GOTERM_MF_DIRECT | GO:0031730~CCR5 chemokine receptor binding | 0.0487133 | 39.876571 | 0.999941 | 46.38536 |
| GOTERM_MF_DIRECT | GO:0005525~GTP binding | 0.055808 | 2.5508512 | 0.999986 | 51.16814 |
| GOTERM_MF_DIRECT | GO:0005509~calcium ion binding | 0.0614051 | 1.996681 | 0.999996 | 54.6609 |
| GOTERM_MF_DIRECT | GO:0004252~serine-type endopeptidase activity | 0.0646724 | 3.2916981 | 0.999998 | 56.59201 |
| GOTERM_MF_DIRECT | GO:0016787~hydrolase activity | 0.0767985 | 1.5477208 | 1 | 63.11659 |
| GOTERM_MF_DIRECT | GO:0030246~carbohydrate binding | 0.0806927 | 3.0473362 | 1 | 65.01216 |

**Table S2. Primers used in this study**

| GENE | PRIMER SEQUENCE (5'-3'） | |
| --- | --- | --- |
| CCL2 | CAGCCAGATGCAATCAATGCC |  |
| CCL7  CCL12 | TGGAATCCTGAACCCACTTCT  GCTGCTTTCAGCATCCAAGTG  CCAGGGACACCGACTACTG  ATTTCCACACTTCTATGCCTCCT  ATCCAGTATGGTCCTGAAGATCA | |
| CCL8 | TGCTGAAGCTCACACCCTTG | |
|  | GGAATGGAAACTGAATCTGGCTG | |
| CXCL3 | TGCATCAGTGACGGTAAACCA | |
|  | TTCTTCAGCCGTGCAACAATC | |
| IL-23 | ATGCTGGATTGCAGAGCAGTA | |
|  | ACGGGGCACATTATTTTTAGTCT | |
| IL-1α | GCACCTTACACCTACCAGAGT | |
|  | AAACTTCTGCCTGACGAGCTT | |
| IFN-α | ATGAACGCTACACACTGCATC | |
|  | CCATCCTTTTGCCAGTTCCTC | |
| TNF-α | CCCTCACACTCAGATCATCTTCT | |
|  | GCTACGACGTGGGCTACAG | |
| IL-12 | TGGTTTGCCATCGTTTTGCTG | |
|  | ACAGGTGAGGTTCACTGTTTCT | |
| IL-1β | GCAACTGTTCCTGAACTCAACT | |
|  | ATCTTTTGGGGTCCGTCAACT | |
| IL-10 | GCTCTTACTGACTGGCATGAG | |
|  | CGCAGCTCTAGGAGCATGTG | |
| IL-6 | TAGTCCTTCCTACCCCAATTTCC | |
|  | TTGGTCCTTAGCCACTCCTTC | |
| IL-27 | CTGTTGCTGCTACCCTTGCTT | |
|  | CACTCCTGGCAATCGAGATTC | |
| IL-17 | TTTAACTCCCTTGGCGCAAAA | |
|  | CTTTCCCTCCGCATTGACAC | |
| IFN-β | CAGCTCCAAGAAAGGACGAAC | |
|  | GGCAGTGTAACTCTTCTGCAT | |
| GM-CSF | GGCCTTGGAAGCATGTAGAGG | |
|  | GGAGAACTCGTTAGAGACGACTT | |
| MMP2 | CAAGTTCCCCGGCGATGTC | |
|  | TTCTGGTCAAGGTCACCTGTC | |
| MMP9 | CTGGACAGCCAGACACTAAAG | |
|  | CTCGCGGCAAGTCTTCAGAG | |
| MMP12 | GAGTCCAGCCACCAACATTAC | |
|  | GCGAAGTGGGTCAAAGACAG | |
| MMP13 | CTTCTTCTTGTTGAGCTGGACTC | |
|  | CTGTGGAGGTCACTGTAGACT | |
| iNOS | GTTCTCAGCCCAACAATACAAGA | |
|  | GTGGACGGGTCGATGTCAC | |
| CD86 | TGTTTCCGTGGAGACGCAAG | |
|  | TTGAGCCTTTGTAAATGGGCA | |
| Chil3 | CAGGTCTGGCAATTCTTCTGAA | |
|  | GTCTTGCTCATGTGTGTAAGTGA | |
| Retnlg | CTTGCCAATCGAGATGACTGT | |
|  | ACCCAGTAGCAGTCATCCCA | |
| Mrc1 | CTCTGTTCAGCTATTGGACGC | |
|  | CGGAATTTCTGGGATTCAGCTTC | |
| IL-4 | GGTCTCAACCCCCAGCTAGT | |
|  | GCCGATGATCTCTCTCAAGTGAT | |
| CCL3 | TTCTCTGTACCATGACACTCTGC | |
|  | CGTGGAATCTTCCGGCTGTAG | |
| CCL4 | TTCCTGCTGTTTCTCTTACACCT | |
|  | CTGTCTGCCTCTTTTGGTCAG | |
| CCL5 | GCTGCTTTGCCTACCTCTCC | |
|  | TCGAGTGACAAACACGACTGC | |
| CXCL2 | TGTGACGGCAGGGAAATGTA | |
|  | TGCTCTAACACAGAGGGAAACA | |
| CXCL10 | CCAAGTGCTGCCGTCATTTTC | |
|  | GGCTCGCAGGGATGATTTCAA | |
| CXCL12 | TGCATCAGTGACGGTAAACCA | |
|  | TTCTTCAGCCGTGCAACAATC | |
| GAPDH | AGGTCGGTGTGAACGGATTTG | |
|  | TGTAGACCATGTAGTTGAGGTCA | |

**Table S3. Antibodies used in this study**

| MARKER (SPECIES) | DILUTION | DISTRIBUTOR/SOURCE  (CATALOGUE NUMBER) |
| --- | --- | --- |
| Primary antibodies |  |  |
| CD11b/ITGAM Rabbit pAb | 1:100 | Zen-Bio (380675) |
| MCP1/CCL2 (5H2) Mouse mAb | 1:1000 (WB) | Zen-Bio (220691) |
|  | 1:200 (IF) |  |
| TNFA Rabbit Ab | 1:500 (WB)  1:100 (IF) | Affinity (AF7014) |
| IL1B Rabbit Ab | 1:1000 (WB)  1:100 (IF) | Affinity (DF6251) |
| CD4 Rabbit Ab | 1:100 (IF) | Zen-Bio (500363) |
| β-Tubulin Mouse mAb (4F2) | 1:5000 | EMAR (EM31013) |
| Rabbit IgG Isotype Control Rabbit pAb | 1:100 | Zen-Bio (380763) |
| Mouse IgG Isotype Control | 1:100 | GeneTex (GTX35009) |
|  |  |  |
| Secondary antibodies  Anti-mouse IgG HRP-linked Ab  Anti-rabbit IgG HRP-linked Ab  Anti-rabbit IgG (Alexa Fluor® 488 Conjugate)  Anti-mouse IgG (Alexa Fluor® 488 Conjugate) | 1:5000  1:5000  1:500  1:500 | CST (7076)  CST (7074)  CST (4412)  CST (4408) |
| Antibodies used in flow cytometry |  |  |
| NK1.1-PE Ab | 1:100 | BioLegend (108707) |
| CD3-PE Ab | 1:100 | BioLegend (100205) |
| CD19-PE Ab | 1:100 | BioLegend (115507) |
| Ly6G-PE Ab | 1:100 | BioLegend (127607) |
| CD11b-PE/CY7 Ab | 1:100 | BioLegend (101215) |
| Ly6C-APC Ab | 1:100 | BioLegend (128015) |
| CD45-PE/CY7 Ab | 1:100 | BioLegend (103113) |
| F4/80-BV421 Ab | 1:100 | BioLegend (123131) |
| TNF-α-FITC Ab | 1:800 | Invitrogen (11-7321-81) |
| IL-1β-FITC Ab | 1:200 | Invitrogen (11-7018-42) |
| Isotype Ctrl-PE Ab | 1:100 | BioLegend (400607) |
| Isotype Ctrl- PE/CY7 Ab | 1:100 | BioLegend (400617) |
| Isotype Ctrl- APC Ab | 1:100 | BioLegend (400713) |
| Isotype Ctrl-BV421 Ab | 1:100 | BioLegend (400535) |
